# Convergent evolution of elicitin perception by divergent pattern-recognition receptors

**DOI:** 10.1093/plcell/koad003

**Published:** 2023-01-13

**Authors:** Thomas A DeFalco

**Affiliations:** Assistant Features Editor, The Plant Cell, American Society of Plant Biologists, USA; Department of Biology, Western University, London, ON, N6A 5B7, Canada

Plants possess a sophisticated immune system that operates at the cellular level. The first layer of this immune system relies on pattern-recognition receptors (PRRs), plasma membrane-localized proteins that perceive extracellular elicitor molecules (e.g. pathogen-associated molecular patterns, PAMPs) to activate so-called pattern-triggered immunity (PTI). Many PRRs have been described in the previous two decades, all of which are receptor kinases (RKs) or receptor proteins (RPs) (Albert et al., 2020). Elicitor perception by PRRs activates a cascade of well-described molecular signaling events (DeFalco and Zipfel, 2021), including reactive oxygen species production, calcium ion fluxes, membrane depolarization, MAP kinase cascades, large-scale transcriptional reprogramming, and, in some cases, cell death.

The oomycete genus *Phytophthora* (from the Greek, “the plant destroyer”) includes some of the most devastating plant pathogens, such as *P. infestans*, the causal agent of 19th-century European potato famines. *Phytophthora* species secrete elicitins (ELIs), small proteins characterized by a conserved cysteine-rich elicitin domain, that function in sterol acquisition to promote *Phytophthora* growth, as well as related ELI-like proteins (ELLs) that have less conserved elicitin domains and divergent C-terminal extensions (Derevnina et al., 2016). Several Solanaceous plant species also perceive ELIs as PAMPs to induce PTI and cell death, including wild species of both potato (*Solanum microdontum*) and tobacco (*Nicotiana benthamiana*). In *S. microdontum*, the ELI receptor was previously identified as the leucine-rich repeat (LRR)-RP ELICITIN RECEPTOR (ELR) (Du et al., 2015). However, ELR is not widely conserved and is absent in *N. benthamiana*, begging the question: what is the molecular identity of the ELI receptor in this species?

In the current issue, Zhaodan Chen and colleagues (Chen et al., 2023) directly addressed this question by individually silencing members of the LRR-RK and LRR-RP families in *N. benthamiana* and monitoring cell death in response to INF1, a well-characterized ELI from *P. infestans*. Cell death in response to INF1 was specifically compromised upon silencing a single previously uncharacterized LRR-RP, which the authors named RESPONSE TO ELICITINS (REL). Further biochemical and genetic assays confirmed that REL acts as a *bona fide* ELI receptor that directly recognizes multiple ELIs (in addition to INF1) and ELLs to trigger immune signaling and cell death. Furthermore, heterologous expression of REL in pepper (*Capsicum annuum*) conferred both ELI-induced cell death and increased resistance to *P. capsici* infection, indicating that this immune receptor can be successfully deployed in other Solanaceous species to bolster immunity against *Phytophthora* (Chen et al., 2023).

Interestingly, *N. benthamiana* REL is phylogenetically divergent from *S. microdontum* ELR (Chen et al., 2023), indicating convergent evolution of ELI perception by these two Solanaceous species (see Figure 1). Concomitantly, the authors used targeted mutagenesis of INF1, ELR, and REL to determine that ELR and REL recognize ELIs via distinct molecular mechanisms involving specific domains. Further studies will be required to fully resolve the structural and evolutionary basis for these two ELI recognition mechanisms. This work highlights the diversity of immune receptors that can exist even among closely related species and underscores the potential applications of such receptors in improving disease resistance in crops.

**Figure 1:**
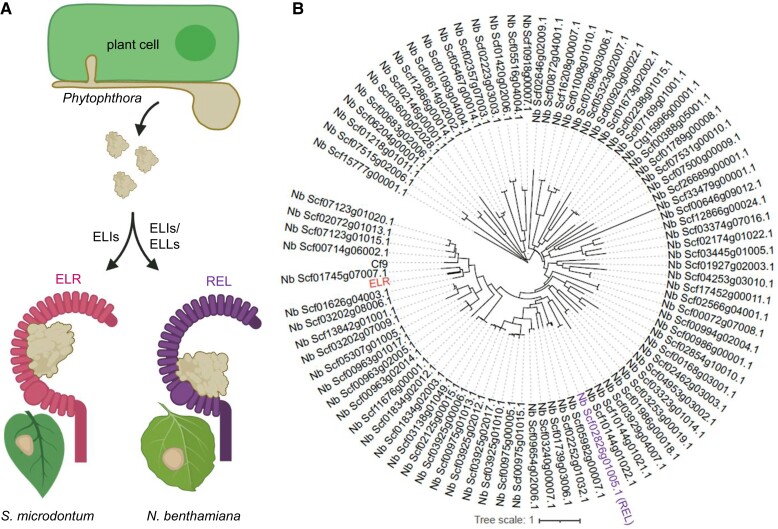
Evolution of elicitin perception. A, Distinct elicitin perception in Solanaceous species via convergent evolution. Created with BioRender.com (B) Phylogenetic tree showing the relationship between *S. microdontum* ELR and *N. benthamiana* LRR-RPs. Adapted from Chen et al. (2023), Figure 6A.

## References

[koad003-B1] Albert I , Hua C, Nürnberger T, Pruitt RN, Zhang L (2020) Surface sensor systems in plant immunity. Plant Physiol 182(4): 1582–1596 31822506 10.1104/pp.19.01299 PMC7140916

[koad003-B2] Chen Z , Liu F, Zeng M, Wang L, Liu H, Sun Y, Wang L, Zhang Z, Chen Z, Xu Y, et al (2023) Convergent evolution of immune receptors underpins distinct elicitin recognition in closely related Solanaceous plants. Plant Cell 35 (4): 1186–1201 10.1093/plcell/koad002 PMC10052394 36625683

[koad003-B3] DeFalco TA , Zipfel C (2021) Molecular mechanisms of early plant pattern-triggered immune signaling. Mol Cell 81(17): 3449–3467 34403694 10.1016/j.molcel.2021.07.029

[koad003-B4] Derevnina L , Dagdas YF, De la Concepcion JC, Bialas A, Kellner R, Petre B, Domazakis E, Du J, Wu C-H, Lin X, et al (2016) Nine things to know about elicitins. New Phytol 212(4): 888–895 27582271 10.1111/nph.14137

[koad003-B5] Du J , Verzaux E, Chaparro-Garcia A, Bijsterbosch G, Keizer LCP, Zhou J, Liebrand TWH, Xie C, Govers F, Robatzek S, et al (2015) Elicitin recognition confers enhanced resistance to Phytophthora infestans in potato. Nat Plants 1(4): 15034 27247034 10.1038/nplants.2015.34

